# Malignant Transformation of Endometriosis in the Ischioanal Fossa

**DOI:** 10.1155/2018/5643040

**Published:** 2018-04-23

**Authors:** Jordan S. Klebanoff, Shivani K. Shah, Mark G. Cadungog

**Affiliations:** ^1^Department of Obstetrics & Gynecology, Christiana Care Health System, Newark, DE, USA; ^2^Division of Gynecologic Oncology, Christiana Care Health System, Newark, DE, USA

## Abstract

We present the case of a 28-year-old nulliparous female with malignant transformation of an ectopic focus of endometriosis in the right ischioanal fossa. A 28-year-old nulliparous patient with a past medical history of polycystic ovarian syndrome (PCOS) was diagnosed with endometrioid adenocarcinoma in her right ischioanal fossa. Initially, patient presented to an emergency department and underwent a CT scan of the appendix to rule out appendicitis. A multiloculated cystic lesion adjacent to the right obturator internus muscle was found. She underwent surgical resection of the mass, which confirmed FIGO grade 2 endometrioid adenocarcinoma, followed by localized radiation therapy. Malignancy arising in endometriosis is rare, and the influence of PCOS on the rate of malignant transformation is not well established.

## 1. Introduction

Endometriosis is a common clinical dilemma affecting up to 10% of reproductive aged women, with an even higher incidence in women with pelvic pain [1]. In the majority of women endometriosis involves the ovaries, fallopian tubes, uterosacral ligaments, and pelvic peritoneum [2]. However, there is evidence of endometriosis involving organs and tissues outside of the pelvis with the abdominal wall being the most common extrapelvic site of involvement [3]. Clinically, endometriosis displays certain behaviors similar to malignancies, including invasion into local tissue and distant spread. Despite this behavior, endometriosis is considered to be a benign disorder. Deep infiltrating implants have been found to contain mutations in well-known cancer-driver genes [4]. In this case, we present a patient found to have endometrioid adenocarcinoma arising in a focus of extrapelvic endometriosis.

## 2. Case

This is a 28-year-old nulliparous patient with a past medical history of polycystic ovarian syndrome (PCOS), anxiety, and depression with newly diagnosed endometrioid adenocarcinoma in her right ischioanal fossa. The patient originally presented to the emergency department due to increasing right lower quadrant and back pain concerning appendicitis. She underwent a CT of the abdomen and pelvis which showed “no evidence of acute inflammatory changes with the abdomen and pelvis. A 4.0 × 2.4 cm multi-loculated cystic lesion adjacent to the right obturator internus muscle, medically indeterminate.”

She was referred to a general surgeon and underwent an MRI of the abdomen and pelvis that revealed a “partially enhancing well-circumscribed multilobulated heterogeneous right inferior pelvic neoplasm, contacting the posteroinferior aspect of the right obturator internus and extending into the right ischioanal fossa.” Due to the inability to definitively characterize the lesion the decision was made to proceed with upper endoscopy, colonoscopy, and a CT guided fine needle aspiration. The patient's endoscopic procedures were unremarkable; however, the biopsy revealed “low grade adenocarcinoma, FIGO grade 1-2, endometrioid carcinoma.”

Given the new finding of an ectopic endometrial cancer, she was subsequently referred to a Gynecologic Oncologist for further management. The patient underwent an in-office pelvic exam that was unremarkable, as well as an endometrial biopsy. Her endometrial biopsy showed no evidence of intrauterine pathology. The case was discussed at the institution's tumor board and the patient's images were reviewed. There was no visible evidence of intra-abdominal disease based on the patient's CT scan and MRI. Ultimately, the decision was made to proceed with surgery followed by a consult to discuss radiation therapy with the institution's Radiation Oncologist. The patient underwent a radical resection of the right perirectal soft tissue malignancy as well as an exam under anesthesia, dilation and curettage, and laparoscopic ovarian transposition using robotic assistance.

The final surgical pathology was consistent with “well differentiated FIGO grade 2 endometrioid adenocarcinoma arising within a cystic focus, compatible with endometriosis.” Surgical margins were negative and on the uterine curettage specimen there was “no evidence of hyperplasia or malignancy identified.” The patient returned to the hospital on postoperative day number three and was diagnosed with pneumonia. She was treated with antibiotics and ultimately discharged home in stable condition on hospital day number two with outpatient follow-up in the office.

The patient had a postoperative CT scan that was reviewed by a Radiation Oncologist who felt that the ovaries were outside of the planned radiation field. The patient expressed a strong interest in maintaining the potential for future fertility; though given her known history of PCOS she was counseled about hormonal contraceptive regimens until she is trying to conceive. The patient underwent a 25-fraction course of radiation to the right perirectal region. She is scheduled to undergo repeat imaging in 3 months following her radiation treatment for evaluation for any evidence of local recurrence.

## 3. Discussion

Endometriosis is considered a benign disease process characterized by ectopic inflammatory estrogen-dependent lesions composed of endometrial glands and stroma found outside of the uterine cavity [4]. Polycystic ovary syndrome has an estimated prevalence of up to 21% in reproductive aged women. The altered endocrine and metabolic environments in women with PCOS are believed to increase their risk of certain cancer types. An association between PCOS and endometrial cancer has been well established [5]. The majority of identified endometrial malignancies that are believed to be related to PCOS are endometrioid adenocarcinomas [6].

There are several prevailing theories of the pathophysiology of endometriotic lesions which include the coelomic metaplasia theory, the embryonal rest theory, and the retrograde menstruation theory [7]. The first two theories both suggest cellular transformations in either the Wolffian duct system or coelomic mesothelium but can only account for lesions in close proximity to the pelvic organs. The retrograde menstruation theory has been linked to local disease as well as distant intraabdominal spread and intrathoracic spread. None of these leading theories would explain an ectopic focus of endometriosis within the ischioanal fossa.

Malignant transformation of endometriosis is a phenomenon well described in the literature with varying reviews and case reports published [8]. Establishing the exact incidence of malignant transformation of endometriosis is difficult; however, it has been estimated to occur in 0.6–0.8% of women with ovarian endometriosis [9]. Our review of the literature found no described cases of ischioanal fossa endometriosis, nor did we find any evidence regarding the incidence of malignant transformation of endometriosis in this anatomical site. There are published reports of perineal endometriosis; however, these cases are described after vaginal birth with or without episiotomy [2]. We present the case of a nulliparous female where these theories do not apply.

Genomewide association studies have linked certain genetic markers with an increased risk of endometriosis [10]. Recent data have shown that benign deep infiltrating endometriosis shared somatic mutations with well-established cancer-driver genes [4]. This study also found that nonovarian deep infiltrating endometriosis, even when containing cancer-associated mutations, rarely transformed into cancer. This study is limited by its small size including endometriotic lesions of only 27 women and did not account for the presence of comorbidities including PCOS.

We described the case of a young nulliparous female with no known history of endometriosis that presumably underwent a malignant transformation of endometriosis located in the ischioanal fossa. She underwent fertility sparing surgery, as she had no evidence of intrauterine disease. She had a laparoscopic ovarian transposition to remove her ovaries from the planned radiation field and then underwent 25 fractions of radiation therapy. She was counseled about hormonal contraception and will undergo surveillance visits and postoperative imaging for evaluation for any signs of disease recurrence. Despite the abundance of evidence linking PCOS to uterine cancer, no data exist regarding whether or not there is an increased rate of malignant transformation of endometriosis in women diagnosed with PCOS. Further research is needed to determine if women with endometriosis and PCOS are more likely to develop ectopic foci of endometrial cancer.

## Supplementary Materials

Supplementary Materials Figure 1: MRI demonstrating a partially enhancing well-circumscribed multilobulated heterogeneous right inferior pelvic neoplasm, contacting the posteroinferior aspect of the right obturator internus and extending into the right ischioanal fossa. Figure 2: CT guided fine needle aspiration of the multiloculated cystic lesion adjacent to the right obturator internus muscle. Figure 3: sections show malignant appearing cells with enlarged irregular nuclei with a cribriform growth pattern and interspersed acute inflammatory cells. The immunohistochemical staining pattern (not shown) was consistent with endometrioid endometrial adenocarcinoma. Figure 4: CT imaging following robotic-assisted laparoscopic ovarian transposition showing the ovaries elevated out of the pelvis, lateral to the psoas muscles, marked by surgical clips.
