# Supplementary material for: Malignant Transformation of Endometriosis in the Ischioanal Fossa
Source: Case Rep Obstet Gynecol. 2018 Apr 23;2018:5643040. doi: 10.1155/2018/5643040 (PMC5937550; doi:10.1155/2018/5643040)
Supplement: Supplementary Materials — Figure 1: MRI demonstrating a partially enhancing well-circumscribed multilobulated heterogeneous right inferior pelvic neoplasm, contacting the posteroinferior aspect of the right obturator internus and extending into the right ischioanal fossa. Figure 2: CT guided fine needle aspiration of the multiloculated cystic lesion adjacent to the right obturator internus muscle. Figure 3: sections show malignant appearing cells with enlarged irregular nuclei with a cribriform growth pattern and interspersed acute inflammatory cells. The immunohistochemical staining pattern (not shown) was consistent with endometrioid endometrial adenocarcinoma. Figure 4: CT imaging following robotic-assisted laparoscopic ovarian transposition showing the ovaries elevated out of the pelvis, lateral to the psoas muscles, marked by surgical clips. [file 5643040.f1.docx]

Figure 1


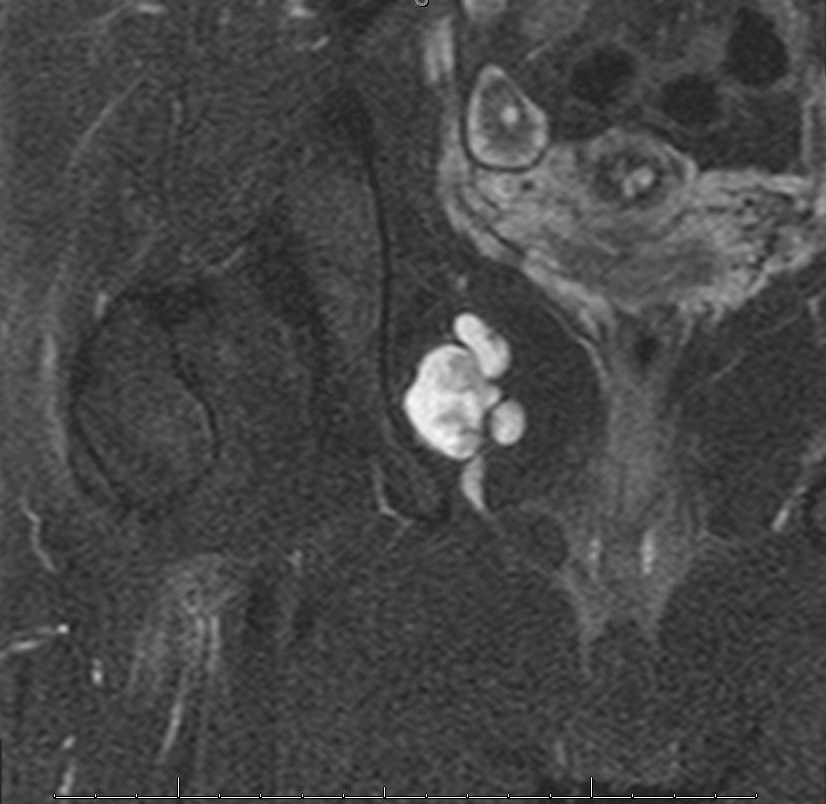


MRI demonstrating a partially enhancing well-circumscribed multi-lobulated heterogeneous right inferior pelvic neoplasm, contacting the postero-inferior aspect of the right obturator internus and extending into the right ischioanal fossa.

Figure 2


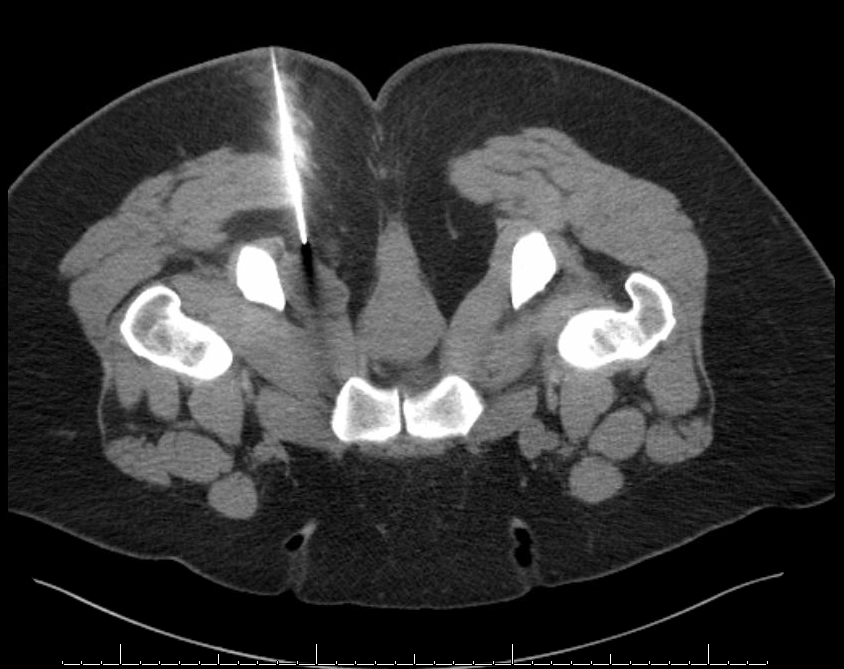


CT guided fine needle aspiration of the multi-loculated cystic lesion adjacent to the right obturator internus muscle

Figure 3


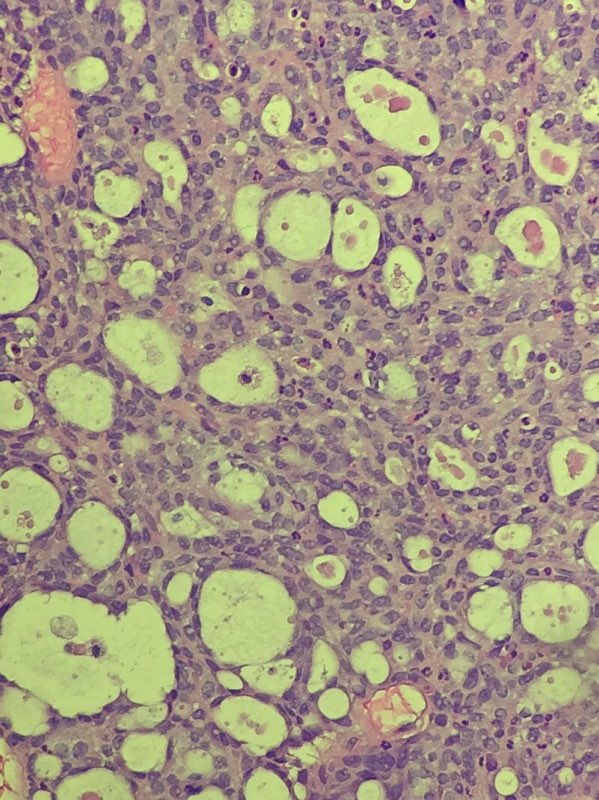


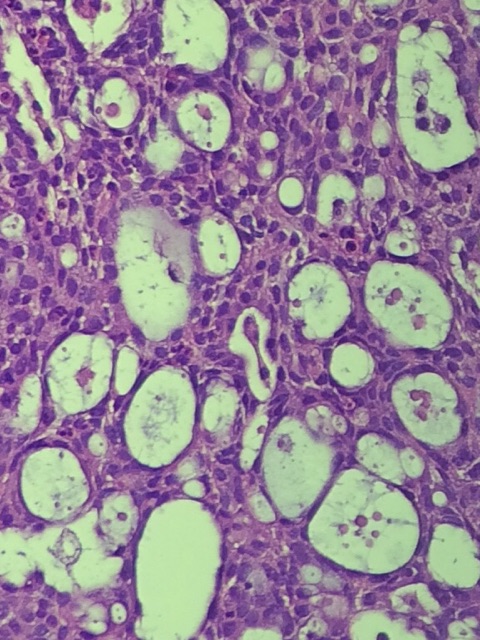


Sections show malignant appearing cells with enlarged irregular nuclei with a cribiform growth pattern and interspersed acute inflammatory cells. The immunohistochemical staining pattern (not shown) was consistent with endometrioid endometrial adenocarcinoma.

Figure 4


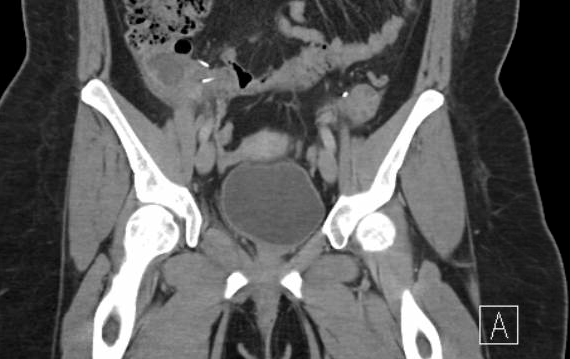


CT imaging following robotic-assisted laparoscopic ovarian transposition showing the ovaries elevated out of the pelvis, lateral to the psoas muscles, marked by surgical clips.
